# Breast Cancer Profile among Patients with a History of Chemoprevention

**DOI:** 10.1155/2016/9216375

**Published:** 2016-12-18

**Authors:** Freya R. Schnabel, Sarah Pivo, Jennifer Chun, Shira Schwartz, Ana Paula Refinetti, Deborah Axelrod, Amber Guth

**Affiliations:** ^1^Department of Surgery, New York University Langone Medical Center, New York, NY, USA; ^2^School of Medicine, New York University Langone Medical Center, New York, NY, USA; ^3^Department of Breast Surgical Oncology, The University of Texas MD Anderson Cancer Center, Houston, TX, USA

## Abstract

*Purpose*. This study identifies women with breast cancer who utilized chemoprevention agents prior to diagnosis and describes their patterns of disease.* Methods*. Our database was queried retrospectively for patients with breast cancer who reported prior use of chemoprevention. Patients were divided into primary (no history of breast cancer) and secondary (previous history of breast cancer) groups and compared to patients who never took chemoprevention.* Results*. 135 (6%) of 2430 women used chemoprevention. In the primary chemoprevention group (*n* = 18, 1%), 39% had completed >5 years of treatment, and fully 50% were on treatment at time of diagnosis. These patients were overwhelmingly diagnosed with ER/PR positive cancers (88%/65%) and were diagnosed with equal percentages (44%) of IDC and DCIS. 117 (87%) used secondary chemoprevention. Patients in this group were diagnosed with earlier stage disease and had lower rates of ER/PR-positivity (73%/65%) than the nonchemoprevention group (84%/72%). In the secondary group, 24% were on chemoprevention at time of diagnosis; 73% had completed >5 years of treatment.* Conclusions*. The majority of patients who used primary chemoprevention had not completed treatment prior to diagnosis, suggesting that the timing of initiation and compliance to prevention strategies are important in defining the pattern of disease in these patients.

## 1. Introduction

Hormonal therapy has been used in the treatment of hormone sensitive breast cancer for many years. The selective estrogen receptor modulator (SERM) tamoxifen was first introduced in the late 1970s. Initially, tamoxifen was used as part of adjuvant treatment for estrogen receptor positive breast cancer in order to prevent systemic spread of disease [1]. However, evidence accumulated demonstrating the additional benefit of tamoxifen in reducing second episodes of breast cancer. Two trials, the Stockholm trial and the National Surgical Adjuvant Breast Bowel Project (NSABP) B-14 trial, showed that the use of tamoxifen was effective in reducing the incidence of contralateral and ipsilateral second cancers by 50% with improvements in disease-free and overall survival in these patients [2, 3]. The use of agents following an incidence of breast cancer to prevent a second occurrence may be termed secondary chemoprevention of breast cancer.

The identified benefit of tamoxifen in secondary chemoprevention prompted further studies to determine the efficacy of the drug for prevention of disease in high risk women who had no personal history of breast cancer. The use of a chemopreventive agent to prevent breast cancer in patients who have not had a previous diagnosis of breast cancer is termed primary chemoprevention. Studies in the 1990s examined the use of these drugs in patients who were at high risk of developing breast cancer due to a positive family history or a previous biopsy showing breast atypia or lobular carcinoma in situ [4, 5]. The NSABP P-1 prevention trial compared the use of 5 years of tamoxifen with a placebo in high risk women of all ages. The study defined high risk according to the Gail model and used the criterion of the Gail model 5-year risk of greater than 1.7% to define high risk women [6]. This study showed a 50% reduction in the rate of occurrence of invasive and noninvasive breast cancer in patients who had taken tamoxifen, with risk reduction benefits observed for at least 10 years following discontinuation of the drug [4, 5]. The NSABP P-2 STAR trial subsequently compared the efficacy of tamoxifen and raloxifene, two SERMs, as chemopreventive agents in postmenopausal women with increased breast cancer risk. Though the original study showed that raloxifene was only 76% as effective as tamoxifen in preventing primary invasive breast cancer and 78% as effective in reducing the risk of noninvasive breast cancer, the updated study showed that long term use of raloxifene was nearly as effective as tamoxifen. The study showed fewer life-threatening side effects in patients who took raloxifene when compared to tamoxifen (with uterine cancer risk reduction of 0.55 and risk reduction of thromboembolic events of 0.75) [7, 8].

Hormonal therapy for breast cancer has evolved over the years. Specifically, postmenopausal women are also eligible to use aromatase inhibitors (AIs) for endocrine therapy of the disease. In postmenopausal women with breast cancer, the use of AIs when compared to 5 years of tamoxifen alone has been shown to improve disease-free survival and reduce the risk of breast cancer events, including distant recurrence, locoregional recurrence, and contralateral breast cancer [9, 10]. The NCIC CTG MAP.3 trial studied the effect of exemestane, an AI, in preventing breast cancer in high risk postmenopausal women. When compared to placebo, this agent demonstrated a decreased incidence of invasive breast cancer without the increased risk of uterine cancer that was observed in tamoxifen. It is important to note that an increased risk of decreased bone density and arthralgia were also observed with AIs when compared to tamoxifen [11]. The IBIS II trial also compared anastrozole and placebo in high risk women for secondary chemoprevention and found that anastrozole was noninferior to tamoxifen in preventing breast cancer recurrence. Risks of tamoxifen included vasomotor symptoms and deep vein thrombosis whereas anastrozole patients reported fractures, hypercholesterolemia, and stroke. The study concluded that anastrozole was not inferior to tamoxifen but may be preferred in some patients given the difference in side effects of these drugs [12].

At the present time, the drug armamentarium for both primary and secondary chemoprevention of breast cancer is vast and effective and the use of these drugs has become part of the treatment guidelines for high risk patients and patients with previous malignant diagnoses alike [13, 14]. Even with the increased use of these drugs, there is a dearth of current literature available on patients who developed breast cancer despite prior or current use of chemoprevention and the pattern of disease in these patients. The purpose of this study is thus to identify a contemporary cohort of women with newly diagnosed breast cancer who had previously utilized chemopreventive strategies and describe their patterns of disease.

## 2. Methods

The Breast Cancer Database (BCD) is a prospective database that was established in January 2010 at the New York University Langone Medical Center (NYULMC). All patients who are treated for breast cancer at NYULMC are eligible to enroll in the BCD. This database includes information on demographics, family history, previous history of breast biopsies, pathologic characteristics of the tumor, and treatment instituted for the breast cancer (chemotherapy, radiation therapy, and/or hormonal therapy). The BCD was queried for patients who were treated for breast cancer and enrolled in our database during the period from January 2010 to January 2016. Individuals who utilized chemoprevention prior to their current diagnosis were isolated, and this cohort was further divided in two subsets: high risk patients with no previous history of breast cancer (primary chemoprevention group) and patients with previous history of breast cancer (secondary chemoprevention group). We further divided these groups into women who were using chemoprevention at the time of current cancer diagnosis and women who had historically used chemoprevention but were not using it at the time of current diagnosis. We examined the primary and secondary chemoprevention groups separately and included data on the patients who had never taken chemoprevention in order to provide a basis for our comparisons.

Descriptive analyses were used to examine the two groups with regard to the following variables: age, family history, BRCA status, history of biopsy proven atypical hyperplasia (AH) and lobular carcinoma in situ (LCIS), breast cancer histology, stage, estrogen receptor (ER)/progesterone receptor (PR)/HER2/neu status, type of chemopreventive agent used (tamoxifen, raloxifene, or AIs), duration of use, time between primary cancer and subsequent breast malignancy, and location of the breast cancer (contralateral versus ipsilateral). We also examined these variables in women who had not previously used chemopreventive agents in order to establish a baseline for our patient population for these factors. Fisher's Exact Test was used to test for statistically significant relationships between the primary and secondary chemoprevention group, compared to the nonchemoprevention group. All analyses were performed with SAS version 9.3 (SAS Institute Inc., Cary, NC).

## 3. Results

A total of 2430 patients were enrolled in the BCD during the study period. We identified 135 (6%) patients diagnosed with breast cancer who were previous or current users of chemopreventive agents. 18 patients comprised the primary prevention group, and 117 were in the secondary prevention group.  Table 1 contains demographics, risk factors, and tumor characteristics for the study groups.

The risk factor profile of the primary chemoprevention group was found to be significantly different from the nonchemoprevention group. Of patients who were BRCA tested, 3 (60%) patients in our primary chemoprevention group were positive compared with 60 (9%) patients in the nonchemoprevention group (*p* = 0.008). Our primary chemoprevention group had a significantly higher incidence of AH (44%) and LCIS (22%) than the nonchemoprevention group which had 2% incidence of AH and 1% incidence of LCIS (*p* < 0.0001 and *p* < 0.0001). Though not statistically significant, our primary chemoprevention group had a much higher incidence of DCIS (44%) than the secondary or nonchemoprevention groups (27%/23% resp.) and correspondingly lower incidence of IDC (44%) than the secondary or no chemoprevention groups (57%/62%). Though 39% of the primary group and only 25% of the nonchemoprevention group had a family history of breast cancer, this was not found to be statistically significant (*p* = 0.18).

Interestingly, in the primary chemoprevention group, 77% of patients were diagnosed with stage 0 or stage I breast cancer. This was not significantly different from the nonchemoprevention group (*p* = 0.61). In the secondary chemoprevention group 81% of patients were diagnosed with stage 0 or stage 1 breast cancer. When compared to the nonchemoprevention group, the secondary chemoprevention cohort had earlier stage disease (*p* = 0.02).

There was no difference in ER/PR status between the primary chemoprevention group and the nonchemoprevention group. However, when compared to the nonchemoprevention group, the secondary chemoprevention group had significantly fewer ER/PR positive cancers (*p* = 0.004). Though not significant, the rate of triple negative breast cancers was higher in the secondary chemoprevention group (10%) compared to the nonchemoprevention group (7%) (*p* = 0.28).

The type, duration, and timing of the chemoprevention in our primary and secondary groups are included in Table 2. The primary group had higher rates of raloxifene usage (56%) than tamoxifen (39%) or AI (5%). The secondary group had higher rates of tamoxifen usage (70%) than raloxifene (5%) or AI (25%). The primary group took chemoprevention for a shorter time period than the secondary group with 61% of the primary group taking chemoprevention for <5 years and only 27% of the secondary taking chemoprevention for <5 years. Of note, half of the primary group was on chemoprevention at the time of breast cancer diagnosis, compared with 24% of the secondary group.

## 4. Discussion

We found that the primary and secondary chemoprevention groups presented with earlier stage breast cancer than the nonchemoprevention group. This difference achieved statistical significance for the secondary group. This finding likely reflects an increased commitment to screening and surveillance in this patient population.

Currently available chemoprevention agents decrease the risk of ER/PR positive cancers. However, it is interesting to note that the majority of secondary chemoprevention patients still had ER/PR positive disease. Though their history of use of chemoprevention narrows the spectrum of agents available to these patients, a broad menu of endocrine therapies is still available. For example, a 2016 meta-analysis by Graham et al. examines the use of fulvestrant for patients with advanced breast cancer and showed positive greater time to recurrence and decreased metastases with this agent [15].

A finding more concerning than the prevalence of ER/PR positive cancers in this population is the increased incidence of triple negative cancers. Though our numbers were small impeding statistical significance to this variable, we still found a decrease in percentage of ER positivity in this group. This is consistent with the findings of the IBIS-I breast cancer prevention trial follow-up which showed that patients were more likely to be ER and PR negative following tamoxifen usage, though this trial examined patients who were taking primary rather than secondary chemoprevention [16]. The IBIS-I trial did not find a reduction in mortality benefit from use of chemoprevention and actually found more deaths from breast cancer in the chemoprevention group. This is likely due to the fact that endocrine therapies are ineffective in triple negative cancers and these tumors are associated with poor prognosis [17]. An additional etiology of these findings could be related to the barriers which limit use of and compliance with chemoprevention, including side effects and physician challenges to recruit eligible women [18]. Our findings echo these results in our secondary chemoprevention cohort.

The primary chemoprevention patients had tumor profiles that were identical to our nonchemoprevention cohort. This is likely related to the short duration of chemoprevention in this group. These patients likely did not experience the full benefit of treatment due to the short duration of their therapy, possibly explaining why this group had similar rates of ER positivity to the nonchemoprevention group. This suggests that the timing of initiation of chemoprevention is important for benefits of this therapy to be optimized. In women with AH and LCIS, further research must be performed in order to better understand how imminent the risk of malignancy development is in this patient population.

This study has several limitations since it is a retrospective chart review and we relied frequently on patient-reported data. Additionally, our sample size may limit the validity of our results regarding the primary cohort. In addition, our results may also be subject to recall bias.

## 5. Conclusions

Our cohort of women who used chemoprevention drugs were overwhelmingly diagnosed with early stage breast cancer, likely reflecting their commitment to screening and surveillance. Though our secondary chemoprevention group had lower rates of ER positivity than the nonchemoprevention group, the majority of cancers in all groups were still ER positive. The trend towards increased rate of triple negative cancers in secondary chemoprevention patients is worrisome. It is likely that the duration of hormonal therapy in patients with breast cancer will be extended given the results of the ATLAS trial. Despite our study limitations, we postulate that this may cause an increase in triple negative second malignancies in this population [19]. Finally, the primary chemoprevention cohort had tumor characteristics identical to the nonchemoprevention group likely due to their incomplete course of treatment. We look forward to research efforts to determine the imminence of risk of malignancy development in high risk women in order to further maximize the benefits of chemopreventive agents in this patient population.

## Figures and Tables

**Table 1:** Clinical characteristics.

Variables	Primary (*N* = 18, 1%)	%	*p* value^a^	Secondary (*N* = 117, 5%)	%	*p* value^b^	Control (*N* = 2295, 94%)	%
*Median age (years)*	59 (46–76)		0.39	66 (39–85)		<0.0001	59 (22–95)	
*Family history of breast cancer*								
Yes	7	39	0.17	41	35	0.01	573	25
No	11	61		76	65		1722	75
*Genetic testing*								
Yes	5	38	1.00	59	64	<0.0001	633	40
No	8	62		33	36		944	60
Not reported	5	—		25	—		718	—
*BRCA 1,2*								
Positive	3	60	0.008	6	10	0.82	60	9
Negative	2	40		53	90		573	91
*Atypical hyperplasia (AH)*								
Yes	8	44	<0.0001	6	5	0.02	38	2
No	10	56		111	95		2257	98
*Lobular carcinoma in situ (LCIS)*								
Yes	4	22	<0.0001	1	1	1.00	21	1
No	14	78		116	99		2274	99
*Clinical stage of breast cancer*								
0, I	14	77	0.61	94	81	0.02	1605	70
II, III, IV	4	23		23	19		690	30
*Histology*								
DCIS (including DCIS w/microinv)	8	44		31	27		530	23
IDC	8	44	0.15	67	57	0.64	1437	62
ILC	1	6		13	11		222	10
Other invasive	1	6		6	5		106	5
*Estrogen receptor*								
Positive	16	89	1.00	83	73	0.004	1901	84
Negative	2	11		30	27		349	16
Unknown	0	—		4	—		45	—
*Progesterone receptor*								
Positive	11	65	0.59	68	60	0.01	1615	72
Negative	6	35		45	40		632	28
Unknown	1	—		4	—		48	—
*HER2/neu*								
Positive	1	10	1.00	12	14	0.42	220	13
Negative	9	90		72	86		1501	85
Equivocal	0	0		0	0		39	2
Unknown/NA	8	—		33	—		535	—
*Triple negative breast cancer*								
Yes	1	6	1.00	12	10	0.28	170	7
No	17	94		105	90		2125	93

^a^Fisher's Exact Test for primary versus nonchemoprevention groups.

^b^Fisher's Exact Test for secondary versus nonchemoprevention groups.

**Table 2:** Chemoprevention summary.

Variables	Primary chemoprevention (*N* = 18, 13%)	%	Secondary chemoprevention (*N* = 117, 87%)	%
*Chemoprevention type*				
Tamoxifen	7	39	82	70
Raloxifene	10	56	6	5
AI	1	5	29	25
*Chemoprevention duration*				
<5 years	11	61	32	27
≥5 years	7	39	85	73
*Chemoprevention timing*				
Current	9	50	28	24
Previous	9	50	89	76
